# Does Vitamin D-Binding Protein Predict Response to Vitamin D Supplementation in Term and Preterm Newborns? A Prospective Cohort Study

**DOI:** 10.3390/jcm15134856

**Published:** 2026-06-23

**Authors:** Burcu Cebeci, Mehmet Emin Arvas, Dilek Kurnaz, Hakan Çakır, Derya Büyükkayhan, Murat Elevli

**Affiliations:** 1 Department of Neonatology, Haseki Training and Research Hospital, University of Health Sciences, 34265 Istanbul, Türkiye; drdilekkurnaz@gmail.com (D.K.); drhakancakir@hotmail.com (H.Ç.); derya.buyukkayhan@sbu.edu.tr (D.B.); 2 Department of Pediatrics, Haseki Training and Research Hospital, University of Health Sciences, 34265 Istanbul, Türkiye; eminsavra94@gmail.com (M.E.A.); murat.elevli@sbu.edu.tr (M.E.)

**Keywords:** neonatal vitamin D deficiency, newborn, preterm infant, supplementation, vitamin D, 25-hydroxyvitamin D, vitamin D-binding protein

## Abstract

**Background:** Vitamin D-binding protein (DBP) is the principal carrier of circulating 25-hydroxyvitamin D [25(OH)D] and is independently synthesized by the neonate. Whether neonatal DBP at birth adds predictive value beyond baseline 25(OH)D for supplementation response remains unclear. **Methods:** This single-center prospective cohort study enrolled 101 neonates. Neonates with 25(OH)D < 20 ng/mL (supplementation-response cohort; *n* = 59: 29 preterm, 30 term) received 800 IU/day oral cholecalciferol for 8 weeks; neonates with 25(OH)D ≥ 20 ng/mL served as baseline reference controls (*n* = 42). Serum 25(OH)D and DBP were measured at baseline and week 8 in the supplementation-response cohort. **Results:** Median baseline 25(OH)D was 8.6 [6.7–12.0] ng/mL and median baseline DBP was 4.9 [3.7–8.1] µg/mL. After supplementation, 25(OH)D increased significantly (median Δ = 17.7 ng/mL; *p* < 0.001), with 55/59 (93.2%) achieving sufficiency. In multivariable regression, gestational age was the strongest independent predictor of Δ25(OH)D (β = −0.440, *p* = 0.001), followed by baseline 25(OH)D (β = −0.314, *p* = 0.015); baseline DBP was not significant (β = 0.072, *p* = 0.551). **Conclusions:** Baseline DBP did not independently predict supplementation response. Lower gestational age and lower baseline 25(OH)D were associated with greater increases in 25(OH)D after supplementation, whereas baseline DBP provided no additional predictive value. Supplementation with 800 IU/day for 8 weeks was effective across gestational-age categories. Routine DBP measurement does not appear to provide additional clinical value for guiding neonatal vitamin D supplementation.

## 1. Introduction

Vitamin D plays a critical role not only in skeletal health and mineralization but also in a wide range of biological processes, including cell growth, immunity, anti-inflammatory responses, neurological development, and insulin synthesis, mediated through autocrine and paracrine pathways [1,2,3,4]. Vitamin D deficiency has been associated with various adverse health outcomes, such as rickets, allergic diseases, pregnancy complications, infections, and diabetes [5,6].

At birth and during early postnatal life, neonatal vitamin D status largely reflects the transplacental transfer of maternal circulating 25-hydroxyvitamin D [25(OH)D] to the fetal circulation during pregnancy, although postnatal intake, supplementation, growth, and sun exposure progressively influence circulating 25(OH)D concentrations. Because endogenous vitamin D synthesis is limited in neonates, this maternal transfer plays a pivotal role in determining neonatal vitamin D status [7]. Transplacental transfer is particularly pronounced during the third trimester; however, in cases of preterm birth, this process is interrupted before completion. Consequently, preterm infants are at increased risk of vitamin D deficiency, which may adversely affect their clinical outcomes, which has been associated with fetal growth restriction, fetal distress, and neonatal infections [8]. Risk of vitamin D deficiency is further influenced by feeding type; exclusively breastfed infants are at particular risk, which is insufficient to maintain adequate vitamin D status. Formula-fed infants may partially meet their requirements through fortified formula, although supplementation is still recommended [9,10].

Given the high prevalence of vitamin D deficiency in infancy and its associated risks, routine vitamin D supplementation is widely recommended as standard of care. Current international guidelines, the American Academy of Pediatrics (AAP) [9] and ESPGHAN [10], recommend a daily intake of 400 IU of vitamin D for all infants starting from birth. Higher doses (800–1000 IU/day) are recommended for preterm infants and for those with documented vitamin D deficiency. The Turkish Neonatal Society guidelines similarly recommend 400–800 IU/day depending on clinical status [11].

Vitamin D-binding protein (DBP) is the primary carrier protein, transporting approximately 85% of circulating 25(OH)D in the general population, and plays a central role in the regulation of vitamin D metabolism [12,13]. Notably, neonatal DBP concentrations may differ from adult values owing to immature hepatic synthesis in the newborn period. Both DBP concentration and genotype have been shown to influence levels of biologically active 25(OH)D [14]. According to the free hormone hypothesis, only the unbound fraction of 25(OH)D is biologically active, and DBP modulates this bioavailability [15]. Because DBP does not substantially cross the placenta, neonatal DBP concentrations may reflect endogenous neonatal protein synthesis and postnatal vitamin D transport capacity rather than direct maternal transfer. Despite growing interest in DBP as a modulator of vitamin D bioavailability, it remains unclear whether neonatal DBP levels provide additional predictive value beyond total 25(OH)D concentrations in assessing response to postnatal supplementation, particularly in preterm infants.

We hypothesized that neonatal DBP levels at birth, being independently synthesized by the neonate and not subject to placental transfer, may serve as a predictor of postnatal vitamin D supplementation response. The specific objectives of this study were: (1) to compare baseline serum 25(OH)D and DBP levels between vitamin D–deficient/insufficient term and preterm newborns and vitamin D–sufficient controls; (2) to evaluate changes in 25(OH)D and DBP levels following standardized vitamin D supplementation; (3) to compare the response to vitamin D supplementation between term and preterm newborns within the deficient/insufficient study groups; and (4) to explore whether baseline DBP is associated with post-supplementation 25(OH)D concentrations and the magnitude of 25(OH)D increase after adjustment for clinically relevant baseline factors.

## 2. Materials and Methods

### 2.1. Study Design and Participants

This was a single-center, prospective, controlled observational study with longitudinal follow-up of vitamin D–deficient/insufficient neonates, conducted at the Neonatal Intensive Care Unit of the University of Health Sciences, Haseki Training and Research Hospital, Istanbul, Türkiye. Istanbul is situated in a temperate climate zone with moderate seasonal variation in sunlight exposure, which is an important determinant of vitamin D status. The study was conducted between August and October 2024.

**Figure 1:**
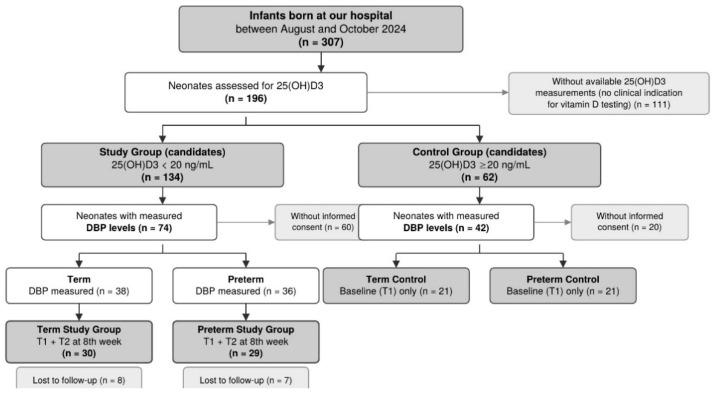
Flow diagram of participant selection and study design.

This study was reported in accordance with the STROBE guidelines. Neonates admitted to the NICU during the study period who underwent clinically indicated serum 25(OH)D testing within the first 24 h of life were screened for eligibility. After baseline assessment, neonates were classified according to serum 25(OH)D status. Those with deficiency or insufficiency, defined as 25(OH)D < 20 ng/mL, constituted the supplementation-response cohort, whereas neonates with sufficient 25(OH)D levels, defined as ≥20 ng/mL, constituted the baseline reference control group. Demographic, prenatal, biochemical, and maternal data of the enrolled neonates were obtained from medical records and the hospital information management system. Preterm neonates were defined as those born before 37 completed weeks of gestation, and term neonates as those born at ≥37 completed weeks. The participant selection process and group allocation are shown in Figure 1.

Inclusion criteria were: gestational age compatible with either term or preterm classification, availability of baseline serum 25(OH)D and DBP measurements within the first 24 h of life. Exclusion criteria were: major congenital anomalies, inborn errors of metabolism, hepatic or renal disease affecting vitamin D metabolism, maternal use of medications known to interfere with vitamin D metabolism, and inability to obtain informed consent.

Neonates with 25(OH)D < 20 ng/mL constituted the supplementation-response cohort, whereas neonates with 25(OH)D ≥ 20 ng/mL served as baseline reference controls. Follow-up 25(OH)D and DBP measurements were obtained only in the supplementation-response cohort.

### 2.2. Vitamin D Supplementation Protocol

In Türkiye, routine vitamin D supplementation of 400 IU/day is recommended for all newborns from the first week of life [11]. For neonates with documented vitamin D deficiency or insufficiency, the Turkish Neonatal Society and ESPGHAN guidelines recommend higher doses (800–1000 IU/day) for an initial treatment period of 8–12 weeks [10,11]. In accordance with these guidelines, neonates in the study group received oral cholecalciferol supplementation at a dose of 800 IU/day, administered as Devit-3 oral drops, initiated within the first postnatal week following baseline (T1) measurements and continued daily for 8 weeks. Neonates in the control group received standard-of-care prophylactic vitamin D supplementation at 400 IU/day; however, because repeat blood sampling was not clinically indicated in vitamin D-sufficient controls, they were not included in longitudinal response analyses. Ethical approval for the study design was granted by the institutional ethics committee, confirming that no neonate was denied standard-of-care supplementation.

### 2.3. Study Outcomes

The primary outcome was the absolute change in serum 25(OH)D from baseline to week 8, calculated as Δ25(OH)D = T2 − T1. Secondary outcomes included week 8 serum 25(OH)D concentration, relative change in 25(OH)D, achievement of vitamin D sufficiency defined as week 8 25(OH)D ≥ 20 ng/mL, change in DBP, and the associations of baseline DBP with Δ25(OH)D and week 8 25(OH)D after adjustment for baseline 25(OH)D and gestational age.

### 2.4. Blood Sampling

In our institution, vitamin D testing is not part of routine universal screening but is performed based on clinical indication. Therefore, the study population represents a clinically indicated cohort rather than a general population sample.

Baseline blood sampling (T1) was performed within the first 24 h of life. Umbilical cord blood was used for blood gas analysis, while venous blood samples were obtained for biochemical analyses (albumin, calcium, phosphorus, magnesium, ALP, CRP), including 25(OH)D and DBP measurements. For neonates with vitamin D deficiency or insufficiency, a follow-up venous blood sample (T2) was obtained at postnatal week 8 (±3 days) during a scheduled outpatient visit. The control group (25(OH)D ≥ 20 ng/mL) was assessed at baseline only, as the primary objective was to evaluate the response to treatment-dose supplementation in vitamin D–deficient/insufficient neonates. Because follow-up blood sampling was not clinically indicated in vitamin D-sufficient controls, the control group was not included in longitudinal treatment response analyses.

### 2.5. Sample Processing

For serum analysis, 1 mL of blood was collected into gel-containing vacuum biochemistry tubes (Sarstedt Monovette, Nümbrecht, Germany) and centrifuged at 3000 rpm for 10 min. Serum 25(OH)D levels were analyzed in the hospital laboratory, and the remaining serum was aliquoted into Eppendorf tubes for DBP analysis. Samples were stored at −80 °C and transported to the laboratory on dry ice (−78.5 °C) after completion of sample collection.

### 2.6. Laboratory Analyses

Serum 25(OH)D concentrations were measured at the hospital ISLAB Laboratory using Siemens Atellica series hormone analyzers (Forchheim, Germany) with commercially available 25(OH)D assay kits (manufacturer-defined reference range: 20–100 ng/mL). The assay predominantly measures 25(OH)D, with limited cross-reactivity for 25(OH)D_2_. As vitamin D supplementation in our setting is exclusively cholecalciferol-based and dietary exposure to ergocalciferol is negligible, measured 25(OH)D levels are considered to closely approximate total circulating 25(OH)D concentrations.

Serum DBP levels were measured using a sandwich ELISA on a Bio-Tek microplate reader with the Sunlong Biotech E1402Hu kit (Hangzhou, China), in accordance with the manufacturer’s standard protocol. DBP concentrations were reported in µg/mL (validated detection range: 0.25–80 µg/mL; expected neonatal range: approximately 3–15 µg/mL). The Sunlong Biotech kit uses polyclonal antibodies; because polyclonal and monoclonal assays may yield different absolute DBP concentrations depending on DBP genotype, comparisons with studies using monoclonal antibody-based assays should be made with caution [16]. Serum samples were kept at +2 °C for 24 h, incubated at 37 °C for 60 min, and diluted with distilled water. A chromogenic substrate was added and incubated for 10 min. The reaction was terminated with a stop solution, and the optical density was measured at 450 nm.

For complete blood count analysis, 0.5 mL of blood was collected into EDTA-containing vacuum tubes (Sarstedt Monovette). Umbilical cord blood gas analyses, including pH, bicarbonate, and base excess, were performed using approximately 1 mL of blood collected into lithium heparin–containing Sarstedt Monovette tubes.

### 2.7. Statistical Analysis

All statistical analyses were performed using IBM SPSS Statistics for Windows, version 26.0 (IBM Corp., Armonk, NY, USA). Figures were generated using Python-based statistical visualization tools (version 3.9; Python Software Foundation, Wilmington, DE, USA). A two-sided *p*-value < 0.05 was considered statistically significant. Because multiple pairwise comparisons were performed (*p*1, *p*2, *p*3) without formal adjustment for multiplicity, borderline significant *p* values should be interpreted with caution.

Continuous variables were assessed for normality using the Shapiro–Wilk test, together with visual inspection of histograms and Q–Q plots. Because several biochemical and hematological variables, particularly DBP, CRP, ALP, WBC, platelet count, and ΔDBP, showed skewed distributions, continuous variables were primarily summarized as median and interquartile range [IQR]. Categorical variables were summarized as numbers and percentages, *n* (%).

Continuous variables were compared using the Mann–Whitney U test. Categorical variables were compared using Pearson’s chi-square test or Fisher’s exact test, as appropriate, according to expected cell counts. When both comparison groups had no events for a categorical variable, no *p*-value was calculated.

For longitudinal analyses, only neonates in the vitamin D supplementation-response cohort were included, because follow-up serum 25(OH)D and DBP measurements were not obtained in vitamin D-sufficient controls. Changes from baseline to week 8 were calculated as follows: Δ25(OH)D = week 8 25(OH)D − baseline 25(OH)D, and ΔDBP = week 8 DBP − baseline DBP. Within-group changes in 25(OH)D and DBP from baseline to week 8 were assessed using the Wilcoxon signed-rank test. The magnitude of change was compared between preterm and term study groups using the Mann–Whitney U test.

Treatment response was defined as achieving serum 25(OH)D ≥ 20 ng/mL at week 8 after supplementation. Responder analyses were performed only as exploratory analyses, because only four neonates failed to achieve vitamin D sufficiency at follow-up. Therefore, multivariable logistic regression was not performed for binary treatment response. Responders and non-responders were compared descriptively using the Mann–Whitney U test for continuous variables and Fisher’s exact test for categorical variables.

Correlation analyses were performed within the supplementation-response cohort. Spearman’s rank correlation coefficient was used to evaluate associations between baseline DBP, baseline 25(OH)D, and supplementation-response parameters, including week 8 25(OH)D, Δ25(OH)D, ΔDBP, and week 8 DBP. Spearman’s correlation was selected because DBP and several response variables were not normally distributed.

Multivariable linear regression analyses were performed to evaluate independent predictors of vitamin D supplementation response. Two prespecified parsimonious models were constructed to minimize overfitting given the modest sample size of the supplementation-response cohort. In Model A, Δ25(OH)D was used as the dependent variable. In Model B, week 8, 25(OH)D concentration was used as the dependent variable. The same core predictors were entered into both models: baseline 25(OH)D, baseline DBP, and gestational age. These variables were selected a priori based on biological relevance and the study hypothesis, rather than solely on univariate statistical significance.

Regression coefficients were reported as unstandardized beta coefficients (B) with 95% confidence intervals, standardized beta coefficients (β), p values, and variance inflation factors (VIFs). Multicollinearity was assessed using VIF, with VIF < 5 considered acceptable. Model performance was summarized using R^2^, adjusted R^2^, and overall model *p*-value. Sensitivity models additionally including maternal vitamin D supplementation during pregnancy and maternal clothing style associated with limited sun exposure were explored to assess the robustness of the main findings.

For graphical presentation, violin plots were used to display baseline 25(OH)D and DBP distributions across the four study groups. Paired line plots were used to illustrate individual-level changes in 25(OH)D from baseline to week 8 in the supplementation-response cohort. Scatter plots with linear regression lines and 95% confidence bands were used to visualize the relationships between baseline DBP and Δ25(OH)D, and between baseline 25(OH)D and Δ25(OH)D. These plots were used for visualization of associations and were interpreted together with the formal correlation and regression analyses.

Follow-up 25(OH)D, follow-up DBP, and treatment-response variables were not applicable to controls and were therefore excluded from longitudinal and response analyses. The number of available observations was reported for each analysis. No imputation was performed.

### 2.8. Sample Size Calculation

The sample size was calculated based on the expected post-supplementation 25(OH)D difference between term and preterm neonates in the study group. Based on published data by Hanson et al. [17], a clinically meaningful difference of 10 ng/mL (SD ± 12 ng/mL) was anticipated. Using a two-sided independent *t*-test with α = 0.05 and a power of 80%, a minimum of 24 participants per group was required. To account for potential dropouts (~20%), at least 29 neonates per study subgroup were targeted. The final sample of 30 term and 29 preterm neonates met this requirement. The control groups (*n* = 21 each) were smaller, which may limit statistical power for certain baseline comparisons. The sample size calculation was performed for the primary group comparison and not specifically for the multivariable regression model; therefore, smaller effect sizes in adjusted analyses may have been underpowered.

## 3. Results

### 3.1. Study Population and Baseline Characteristics

A total of 101 neonates were included in the final analysis. Of these, 59 neonates had vitamin D deficiency or insufficiency and constituted the supplementation-response cohort, whereas 42 neonates had sufficient baseline vitamin D levels and served as baseline reference controls. Baseline demographic and perinatal characteristics are presented in Table 1. Term and preterm study groups differed, as expected, in gestational age, birth weight, Apgar scores, delivery mode, feeding-related variables, and length of hospital stay. Maternal comorbidities and neonatal clinical diagnoses are provided in Table S1.

**Figure 2:**
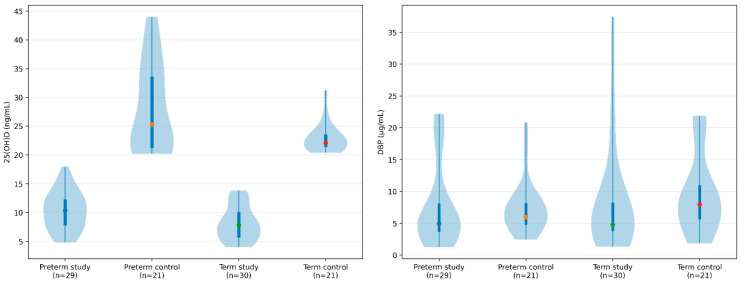
Baseline 25(OH)D and DBP distributions across study groups. Baseline 25(OH)D concentrations by group and baseline vitamin D-binding protein concentrations by group. Violin plots represent the distribution of values. Colored dots indicate the median, and the central vertical bars represent the interquartile range.

Baseline biochemical and hematological parameters are summarized in Table 2. Baseline DBP concentrations did not differ significantly between study and control groups in either preterm or term neonates. The distributions of baseline 25(OH)D and DBP are shown in Figure 2, illustrating a clear separation of 25(OH)D values but substantial overlap of DBP values across groups.

### 3.2. Baseline Vitamin D Status in the Supplementation-Response Cohort

Within the supplementation-response cohort, 43 neonates had vitamin D deficiency (<12 ng/mL) and 16 had vitamin D insufficiency (12–20 ng/mL). Vitamin D deficiency was observed in 18/29 preterm neonates and 25/30 term neonates; this difference did not reach statistical significance (*p* = 0.084).

### 3.3. Response to Vitamin D Supplementation

**Figure 3:**
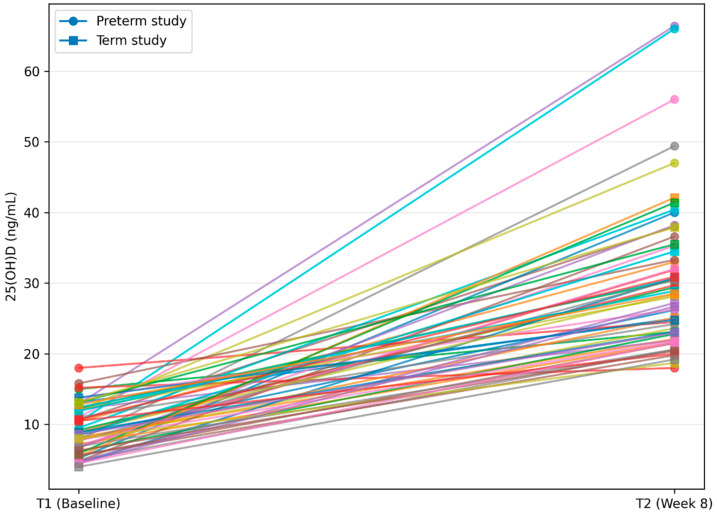
Individual paired changes in serum 25(OH)D from baseline to week 8 in the supplementation-response cohort. Each line represents one neonate. Circles indicate preterm neonates and squares indicate term neonates.

Following 8 weeks of supplementation, serum 25(OH)D concentrations increased significantly in the overall supplementation response cohort and in both preterm and term study subgroups (Table 3). The magnitude of Δ25(OH)D did not differ significantly between preterm and term neonates. DBP levels also increased significantly from baseline to week 8 in the overall cohort and in both subgroups, with no significant difference in ΔDBP between preterm and term neonates. Individual paired changes in 25(OH)D are illustrated in Figure 3. At week 8, 55 of 59 neonates achieved serum 25(OH)D sufficiency, yielding an overall response rate of 93.2% (95% CI, 83.8–97.3%). Response rates were similar in preterm and term neonates. Because only four neonates failed to achieve vitamin D sufficiency, binary treatment-response analyses were considered exploratory and were not used for multivariable logistic regression.

### 3.4. Correlation Analyses

**Figure 4:**
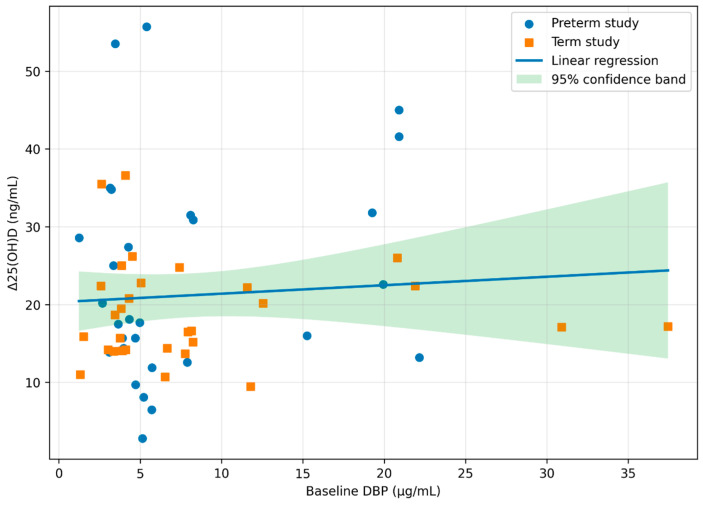
Association between baseline vitamin D-binding protein and change in 25(OH)D after supplementation. Scatter plot showing baseline DBP versus Δ25(OH)D in the supplementation-response cohort. Circles indicate preterm neonates and squares indicate term neonates. The solid line represents the linear regression fit, and the shaded area represents the 95% confidence band.

**Figure 5:**
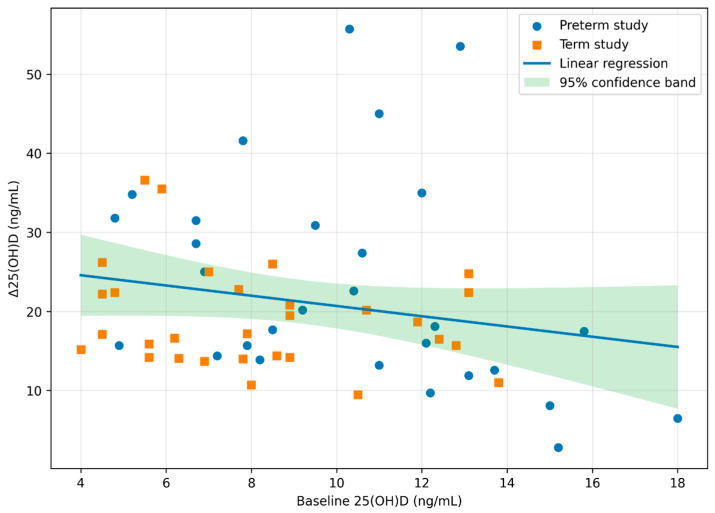
Association between baseline 25(OH)D and change in 25(OH)D after supplementation. Scatter plot showing baseline 25(OH)D versus Δ25(OH)D in the supplementation-response cohort. Circles indicate preterm neonates and squares indicate term neonates. The solid line represents the linear regression fit, and the shaded area represents the 95% confidence band.

Correlations between baseline DBP, baseline 25(OH)D, and supplementation-response parameters are presented in Table 4. Baseline DBP was not significantly correlated with either week 8 25(OH)D or Δ25(OH)D, indicating no meaningful association between baseline DBP and biochemical response to supplementation. These relationships are visualized in Figure 4 and Figure 5. Baseline DBP showed no apparent association with Δ25(OH)D, whereas lower baseline 25(OH)D tended to be associated with a greater increase after supplementation. The latter association should be interpreted cautiously because Δ25(OH)D is mathematically dependent on baseline 25(OH)D, and regression to the mean may partly account for this relationship.

### 3.5. Multivariable Linear Regression Analyses

Multivariable linear regression models are presented in Table 5. In the primary model using Δ25(OH)D as the dependent variable, lower baseline 25(OH)D and lower gestational age were independently associated with a greater increase in 25(OH)D after supplementation, whereas baseline DBP was not independently associated with Δ25(OH)D. In the secondary model using week 8 25(OH)D as the dependent variable, gestational age remained independently associated with week 8 25(OH)D, while baseline 25(OH)D and baseline DBP were not significant predictors. Because week 8 25(OH)D is mathematically related to Δ25(OH)D and baseline 25(OH)D, this secondary model was interpreted as confirmatory. Accordingly, the coefficients for predictors other than baseline 25(OH)D are mathematically identical across models.

In sensitivity models additionally including maternal vitamin D supplementation during pregnancy and maternal clothing style associated with limited sun exposure, the direction and significance of the main findings remained unchanged, and baseline DBP remained non-significant.

### 3.6. Exploratory Responder Analysis

Because only four neonates failed to achieve vitamin D sufficiency at week 8, responder analyses were considered exploratory and are presented in Table S2. Non-responders had lower Δ25(OH)D than responders, whereas baseline 25(OH)D, baseline DBP, gestational age, and birth weight did not differ significantly between groups. Notably, all four non-responders had week 8 25(OH)D values between 18.0 and 19.8 ng/mL, representing marginal failures to reach the 20 ng/mL threshold rather than true treatment resistance. Owing to the very small number of non-responders, no multivariable logistic regression model was constructed.

## 4. Discussion

This prospective cohort study evaluated whether baseline DBP levels at birth provide additional predictive value beyond serum 25(OH)D for determining the response to postnatal vitamin D supplementation in term and preterm newborns. The principal finding was that baseline DBP concentration did not independently predict supplementation response, as assessed by either Δ25(OH)D or week 8 25(OH)D, in univariate or multivariable analyses. In contrast, gestational age emerged as the strongest independent predictor, with preterm neonates demonstrating a greater magnitude of 25(OH)D increase following 8 weeks of 800 IU/day cholecalciferol supplementation. The overall supplementation response rate was high (93.2%), and response rates did not differ between term and preterm neonates.

The lack of an independent association between baseline DBP and supplementation response is a notable negative finding with potential clinical and biological implications. Several mechanistic considerations may explain this observation. First, neonatal DBP is endogenously synthesized primarily by the liver, and hepatic synthetic capacity may not yet be fully mature at birth, particularly in preterm infants [18]. The wide interindividual variability of baseline DBP values observed in our study (range: 1.2–37.4 µg/mL) may partly reflect differences in hepatic maturity. A single measurement at birth may therefore capture a transitional state rather than a stable indicator of vitamin D transport capacity. Second, DBP concentrations are strongly influenced by DBP genotype. The GC gene encodes three major polymorphic variants (GC1F, GC1S, GC2) with differing binding affinities for 25(OH)D [14,16]. Powe et al. [19] reported racial differences in vitamin D and vitamin D-binding protein levels among Americans, likely attributable to differences in the prevalence of common genetic polymorphisms. Enlund-Cerullo et al. [20] demonstrated that GC polymorphisms significantly affect both vitamin D status and the response to supplementation in infants, with certain genotypes associated with lower 25(OH)D concentrations despite identical supplementation regimens. Similarly, Albiñana et al. [21] showed strong genetic correlations between GC variants and both DBP and 25(OH)D levels in neonatal dried blood spots, underscoring that observed DBP concentrations are substantially determined by genotype. Because genotyping was not performed in our study, phenotypic variation in DBP concentration may have obscured genotype-dependent functional differences. Cillero et al. [22] demonstrated that liquid chromatography-tandem mass spectrometry enables phenotyping of DBP isoforms in pediatric populations, suggesting that future studies could benefit from isoform-specific quantification rather than total DBP measurement alone. Third, according to the free hormone hypothesis, it is the unbound (free) fraction of 25(OH)D that is biologically active, and total DBP concentration alone may not adequately capture the relevant parameter driving cellular uptake and response [15]. A further and fundamental consideration is that DBP circulates in substantial molar excess relative to its vitamin D ligands; under physiological conditions, fewer than 5% of DBP binding sites are occupied, and the apo-DBP fraction therefore exceeds 95% [13,16]. Given this large reserve binding capacity, total DBP concentration would not be expected to act as a rate-limiting factor for vitamin D transport or bioavailability except under conditions of severe protein depletion. Outside of marked nutritional protein deficiency or hepatic synthetic failure, DBP concentrations remain relatively stable, and the coexistence of low 25(OH)D with preserved DBP in our cohort is fully consistent with an abundant apo-DBP pool. Our negative finding should therefore be interpreted as empirical confirmation, in a prospective neonatal supplementation cohort, that baseline total DBP does not constrain the 25(OH)D response, rather than as evidence against a role for DBP genotype or free/bioavailable 25(OH)D, which remain unaddressed by total DBP measurement.

These findings are consistent with and extend the limited literature on neonatal DBP. Karras et al. [23] reported that maternal and neonatal DBP levels were not significantly correlated with 25(OH)D status in cord blood, supporting the notion that total DBP is a poor predictor of vitamin D status in neonates. Doneray et al. [24] examined serum DBP in mother-neonate pairs during the lactation period and found that neonatal DBP concentrations were substantially lower than maternal values, consistent with immature hepatic synthesis in the early neonatal period. In adult populations, Björkhem-Bergman et al. [25] demonstrated that DBP concentrations were not affected by high-dose vitamin D supplementation, suggesting that DBP may function as a relatively stable carrier protein that does not dynamically respond to changes in vitamin D supply. This finding aligns with our observation that baseline DBP did not predict the magnitude of 25(OH)D increase. In contrast, Specker et al. [26] reported a negative relationship between DBP and sunshine exposure in exclusively breastfed infants, suggesting a more complex interplay between DBP and environmental determinants of vitamin D status in early life. The present study extends these observations to the supplementation-response context and provides the first prospective evidence that baseline neonatal DBP does not add predictive value for supplementation outcomes in a cohort of deficient and insufficient newborns.

Gestational age was the strongest predictor of supplementation response in the multivariable model, with lower gestational age associated with a greater Δ25(OH)D. Several mechanisms may explain this finding. Tsai et al. [27] recently confirmed that maternal vitamin D status and genetic variants are the primary determinants of cord blood vitamin D levels, reinforcing the importance of the maternal-fetal transfer pathway that is disrupted in preterm birth. Additionally, Vestergaard et al. [28] showed that even high-dose maternal vitamin D supplementation may not fully normalize placental vitamin D metabolism or neonatal vitamin D status, highlighting the physiological constraints of transplacental transfer. Preterm infants also exhibit higher growth velocity during the early postnatal period, which may enhance the metabolic demand for and utilization of supplemented vitamin D.

The observed inverse correlation between baseline 25(OH)D and Δ25(OH)D warrants careful interpretation. Because Δ25(OH)D is calculated by subtracting baseline 25(OH)D from week 8 25(OH)D, this correlation is partly driven by mathematical coupling. Additionally, regression to the mean may contribute neonates selected for low baseline values are expected to show larger increases upon retesting, independent of any intervention effect. The multivariable model partially addresses this by including baseline 25(OH)D as a covariate, but residual confounding cannot be fully eliminated.

Another important finding was the significant increase in DBP concentrations from baseline to week 8 in both term and preterm neonates, which may reflect postnatal maturation of hepatic protein synthesis and increasing vitamin D transport capacity during the early neonatal period [18]. Notably, Björkhem-Bergman et al. [25] found that DBP was unaffected by vitamin D supplementation in adults, suggesting that the DBP increase observed in our neonates is more likely attributable to developmental maturation of hepatic function rather than a direct effect of cholecalciferol supplementation. The moderate inverse correlation between baseline DBP and ΔDBP supports this interpretation, indicating a compensatory catch-up in neonates with initially lower hepatic synthetic capacity.

The overall response rate of 93.2% to 800 IU/day cholecalciferol over 8 weeks confirms the effectiveness of this dosing regimen. Notably, all four non-responders had week 8 25(OH)D between 18.0 and 19.8 ng/mL, representing marginal shortfalls rather than genuine supplementation failure. The comparable response between term and preterm neonates further suggests that this dosing strategy is effective across gestational age categories.

Several strengths of this study merit emphasis. The prospective design with standardized supplementation protocol and uniform follow-up timing reduces confounding by treatment variability. All biochemical measurements were performed using the same assays and instruments, eliminating inter-laboratory variability. The inclusion of both term and preterm neonates in a single study allows direct within-study comparisons. The statistical approach included prespecified models, sensitivity analyses, and transparent reporting of negative findings.

This study has several limitations. The single-center design limits generalizability. DBP genotyping was not performed; given recent evidence that GC polymorphisms substantially affect both DBP concentrations and supplementation response in infants [20,21], genotype-stratified analyses might have revealed associations obscured in the overall cohort. Cillero et al. [22] have demonstrated the feasibility of DBP phenotyping by mass spectrometry in pediatric samples, and incorporating such methods in future studies could provide more informative data than total DBP measurement with immunoassays. Free and bioavailable 25(OH)D were not measured, precluding evaluation of whether DBP-adjusted measures of vitamin D bioavailability predict supplementation response. Follow-up blood samples were not obtained from the control group because repeat sampling was not clinically justified. The sample size was powered for the primary subgroup comparison rather than the regression model. Multiple pairwise comparisons were performed without formal adjustment for multiplicity. The study was conducted during a single season (August–October). Finally, the small number of non-responders (*n* = 4) precluded robust analysis of predictors of treatment failure.

## 5. Conclusions

Baseline DBP concentration at birth did not independently predict response to vitamin D supplementation in either term or preterm newborns with vitamin D deficiency or insufficiency. Gestational age was the strongest independent predictor of supplementation response. Supplementation with 800 IU/day cholecalciferol for 8 weeks achieved vitamin D sufficiency in 93.2% of neonates. These findings suggest that routine DBP measurement does not provide additional clinical value for predicting or guiding vitamin D supplementation outcomes in the neonatal period. Future studies incorporating DBP genotyping, isoform-specific quantification, and measurement of free or bioavailable 25(OH)D are warranted to further clarify the role of DBP in neonatal vitamin D metabolism.

## Figures and Tables

**Table 1:** Baseline demographic and perinatal characteristics of the study population.

Variable	Preterm Study *n* = 29	Preterm Control *n* = 21	Term Study *n* = 30	Term Control *n* = 21	*p*1	*p*2	*p*3
Maternal age, years	28 [26–34]	32 [28–37]	26 [21–29]	29 [26–34]	0.193	0.016	0.017
Gravida	3 [2–4]	2 [1–4]	2 [1–3]	2 [1–2]	0.257	0.211	0.171
Parity	2 [2–3]	2 [1–3]	2 [1–3]	1 [1–2]	0.230	0.134	0.394
Gestational age, weeks	34 [32–35]	34 [33–35]	39 [38–39]	38 [38–39]	0.904	0.033	<0.001
Birth weight, g	2200 [1650–2620]	2205 [1680–2850]	3415 [3042–3535]	3270 [2920–3610]	0.723	0.433	<0.001
Apgar score at 1 min	7 [6–8]	8 [6–9]	9 [8–9]	9 [8–9]	0.387	0.940	0.003
Apgar score at 5 min	8 [7–10]	9 [8–10]	10 [9–10]	10 [8–10]	0.285	0.958	0.007
Umbilical cord pH	7.35 [7.29–7.36]	7.35 [7.29–7.40]	7.33 [7.30–7.35]	7.35 [7.32–7.36]	0.548	0.609	0.542
Umbilical cord lactate, mmol/L	2.20 [1.80–2.90]	2.50 [2.10–3.00]	2.20 [1.82–2.70]	2.30 [1.60–4.00]	0.237	0.901	0.946
Maternal vitamin D supplementation during pregnancy	6 (20.7)	14 (66.7)	7 (23.3)	15 (71.4)	0.001	<0.001	0.807
Maternal limited sun exposure clothing	20 (69.0)	11 (52.4)	23 (76.7)	9 (42.9)	0.233	0.014	0.506
Male sex	19 (65.5)	12 (57.1)	18 (60.0)	15 (71.4)	0.547	0.401	0.661
Cesarean delivery	26 (89.7)	18 (85.7)	13 (43.3)	14 (66.7)	0.686	0.100	<0.001
Formula feeding	10 (34.5)	11 (52.4)	20 (66.7)	9 (42.9)	0.206	0.091	0.013
Total parenteral nutrition	7 (24.1)	10 (47.6)	2 (6.7)	1 (4.8)	0.084	1.000	0.080
Time to full enteral feeding, days	3 [1–6]	3 [1–7]	1 [1–1]	1 [1–3]	0.545	0.325	<0.001
Length of hospital stay, days	11 [6–16]	9 [4–23]	4 [3–6]	6 [3–8]	0.723	0.319	<0.001

Values are presented as median [interquartile range] or *n* (%). *p*1 compares preterm study vs. preterm control; *p*2 compares term study vs. term control; *p*3 compares preterm study vs. term study.

**Table 2:** Baseline biochemical and hematological parameters.

Variable	Preterm Study *n* = 29	Preterm Control *n* = 21	Term Study *n* = 30	Term Control *n* = 21	*p*1	*p*2	*p*3
25(OH)D, ng/mL	10.4 [7.8–12.3]	25.3 [21.2–33.6]	7.8 [5.6–10.1]	22.1 [21.4–23.6]	<0.001	<0.001	0.020
DBP, µg/mL	4.9 [3.6–8.0]	5.9 [4.7–8.1]	4.7 [3.7–8.2]	7.9 [5.6–10.9]	0.198	0.217	1.000
Albumin, g/dL	3.50 [3.10–3.70]	3.60 [3.10–3.70]	3.90 [3.70–4.07]	3.60 [3.30–3.80]	0.775	0.001	<0.001
Calcium, mg/dL	9.50 [9.30–10.00]	9.30 [8.50–9.60]	9.80 [9.33–10.30]	9.50 [9.20–9.90]	0.110	0.212	0.312
Phosphorus, mg/dL	5.70 [5.20–6.50]	5.40 [5.10–6.00]	5.25 [5.00–5.78]	5.00 [4.40–5.50]	0.431	0.162	0.068
Magnesium, mg/dL	1.90 [1.80–2.10]	1.80 [1.70–1.90]	1.90 [1.80–2.00]	1.80 [1.80–1.90]	0.094	0.332	0.236
ALP, U/L	186 [178–224]	166 [153–202]	184 [174–197]	144 [109–158]	0.118	<0.001	0.426
CRP, mg/L	0.50 [0.50–1.00]	0.50 [0.50–0.50]	1.50 [0.50–8.77]	3.20 [1.00–18.00]	0.059	0.247	0.009
WBC, ×10^3^/µL	12.2 [8.8–17.4]	10.2 [8.2–14.0]	14.3 [11.8–19.9]	15.0 [11.2–19.6]	0.298	0.939	0.049
Hemoglobin, g/dL	17.0 [16.0–19.0]	17.5 [16.3–19.0]	17.6 [16.0–19.3]	18.7 [16.3–19.8]	0.637	0.432	0.595
Platelet, ×10^3^/µL	252 [216–314]	276 [189–324]	281 [232–339]	280 [210–319]	0.311	0.373	0.064

Values are presented as median [interquartile range]. *p*1 compares preterm study vs. preterm control; *p*2 compares term study vs. term control; *p*3 compares preterm study vs. term study. DBP, vitamin D-binding protein; ALP, alkaline phosphatase; CRP, C-reactive protein; WBC, white blood cell count.

**Table 3:** Changes in 25(OH)D_3_ and vitamin D-binding protein after supplementation in the study group.

Parameter	Group	T1 Baseline	T2 Week 8	Δ T2–T1	*p*1	*p*2
25(OH)D, ng/mL	Total study group *n* = 59	8.6 [6.7–12.0]	28.4 [22.9–34.9]	17.7 [14.2–25.5]	<0.001	—
25(OH)D, ng/mL	Preterm study *n* = 29	10.4 [7.8–12.3]	30.4 [24.2–38.2]	18.1 [13.9–31.5]	<0.001	0.426
25(OH)D, ng/mL	Term study *n* = 30	7.8 [5.6–10.1]	26.9 [21.6–30.6]	17.1 [14.2–22.4]	<0.001	
DBP, µg/mL	Total study group *n* = 59	4.9 [3.7–8.1]	9.0 [6.5–16.2]	3.0 [0.4–10.0]	<0.001	—
DBP, µg/mL	Preterm study *n* = 29	4.9 [3.6–8.0]	8.2 [6.5–19.6]	3.0 [−0.2–10.2]	0.010	0.897
DBP, µg/mL	Term study *n* = 30	4.7 [3.7–8.2]	9.2 [7.4–14.8]	3.5 [0.9–9.1]	0.002	

*p*1: *p* for within-group change; *p*2: *p* for Δ comparison between preterm and term; Values are presented as median [interquartile range]. Δ was calculated as T2 week 8 value minus T1 baseline value. DBP, vitamin D-binding protein. The *p*-value for the Δ comparison is shown once for each parameter and represents the comparison of Δ values between preterm and term study groups.

**Table 4:** Correlations between baseline DBP, baseline 25(OH)D, and supplementation-response parameters.

Baseline Variable	T2 25(OH)D	Δ25(OH)D	ΔDBP	T2 DBP
Baseline DBP, µg/mL	r = −0.052; *p* = 0.693	r = −0.017; *p* = 0.898	r = −0.524; *p* < 0.001	r = −0.028; *p* = 0.833
Baseline 25(OH)D, ng/mL	r = 0.124; *p* = 0.348	r = −0.281; *p* = 0.031	r = 0.087; *p* = 0.512	r = 0.037; *p* = 0.780

Correlations were calculated in the supplementation-response cohort only. Spearman’s rank correlation coefficient was used. Δ25(OH)D was calculated as week 8 25(OH)D minus baseline 25(OH)D. ΔDBP was calculated as week 8 DBP minus baseline DBP. DBP, vitamin D-binding protein.

**Table 5:** Multivariable linear regression models for vitamin D supplementation response.

Predictor	Model A: Δ25(OH)D B, 95% CI	Standardized β	*p*	VIF	Model B: T2 25(OH)D B, 95% CI	Standardized β	*p*	VIF
Baseline 25(OH)D, ng/mL	−1.004 [−1.806 to −0.201]	−0.314	0.015	1.10	−0.004 [−0.806 to 0.799]	−0.001	0.993	1.10
Baseline DBP, µg/mL	0.102 [−0.240 to 0.445]	0.072	0.551	1.03	0.102 [−0.240 to 0.445]	0.073	0.551	1.03
Gestational age, weeks	−1.257 [−1.968 to −0.546]	−0.440	0.001	1.09	−1.257 [−1.968 to −0.546]	−0.446	0.001	1.09

Model fit: Model A: dependent variable = Δ25(OH)D; R^2^ = 0.221, adjusted R^2^ = 0.178, model *p* = 0.003. Model B: dependent variable = T2 25(OH)D; R^2^ = 0.198, adjusted R^2^ = 0.154, model *p* = 0.007. Model A: dependent variable = Δ25(OH)D. Model B: dependent variable = week 8 25(OH)D. Both models were constructed in the supplementation-response cohort. B represents the unstandardized regression coefficient. β represents the standardized coefficient. CI, confidence interval; DBP, vitamin D-binding protein; VIF, variance inflation factor. Because week 8 25(OH)D equals baseline 25(OH)D plus Δ25(OH)D, and baseline 25(OH)D was included in both models, coefficients for predictors other than baseline 25(OH)D are mathematically expected to be identical across models.

## Data Availability

The datasets used and/or analyzed during the current study are not publicly available due to privacy and ethical restrictions but are available from the corresponding author on reasonable request.

## Supplementary Materials

The following supporting information can be downloaded at: https://www.mdpi.com/article/10.3390/jcm15134856/s1. Table S1: Maternal comorbidities and neonatal clinical diagnoses; Table S2: Characteristics of responders and non-responders in the supplementation-response cohort.

## Abbreviations

The following abbreviations are used in this manuscript:

25(OH)D	25-Hydroxyvitamin D
AAP	American Academy of Pediatrics
ALP	Alkaline Phosphatase
CI	Confidence Interval
CRP	C-Reactive Protein
DBP	Vitamin D-Binding Protein
EDTA	Ethylenediaminetetraacetic Acid
ELISA	Enzyme-Linked Immunosorbent Assay
ESPGHAN	European Society for Paediatric Gastroenterology, Hepatology and Nutrition
GC	Group-Specific Component (Vitamin D-Binding Protein Gene)
IQR	Interquartile Range
NICU	Neonatal Intensive Care Unit
SPSS	Statistical Package for the Social Sciences
STROBE	Strengthening the Reporting of Observational Studies in Epidemiology
VIF	Variance Inflation Factor
WBC	White Blood Cell Count
