# Supplementary material for: Does Vitamin D-Binding Protein Predict Response to Vitamin D Supplementation in Term and Preterm Newborns? A Prospective Cohort Study
Source: J Clin Med. 2026 Jun 23;15(13):4856. doi: 10.3390/jcm15134856 (PMC13360735; doi:10.3390/jcm15134856)
Supplement: Supplementary file 1 [file jcm-15-04856-s001.zip › jcm-4325791-supplementary.pdf]

Table S1. Maternal comorbidities and neonatal clinical diagnoses.

Variable	Preterm study n=29	Preterm control n=21	Term study n=30	Term control n=21	p1	p2	p3
Maternal hypothyroidism	1 (3.4)	1 (4.8)	2 (6.7)	2 (9.5)	1.000	1.000	1.000
Maternal diabetes mellitus	2 (6.9)	4 (19.0)	0 (0.0)	0 (0.0)	0.223	NA	0.237
Maternal hypertension	6 (20.7)	5 (23.8)	1 (3.3)	1 (4.8)	1.000	1.000	0.052
Transient tachypnea of newborn	21 (72.4)	9 (42.9)	3 (10.0)	4 (19.0)	0.035	0.427	<0.001
Hyperbilirubinemia	5 (17.2)	6 (28.6)	22 (73.3)	11 (52.4)	0.491	0.123	<0.001
Sepsis	5 (17.2)	0 (0.0)	12 (40.0)	12 (57.1)	0.066	0.227	0.054
Respiratory distress syndrome	7 (24.1)	4 (19.0)	0 (0.0)	1 (4.8)	0.741	0.412	0.005
Hypoglycemia	1 (3.4)	1 (4.8)	1 (3.3)	1 (4.8)	1.000	1.000	1.000
Patent ductus arteriosus	6 (20.7)	4 (19.0)	0 (0.0)	0 (0.0)	1.000	NA	0.011
Bronchopulmonary dysplasia	2 (6.9)	0 (0.0)	0 (0.0)	0 (0.0)	0.503	NA	0.237
Intravenous hydration	21 (72.4)	12 (57.1)	5 (16.7)	6 (28.6)	0.261	0.327	<0.001

Values are presented as n (%). p1 compares preterm study vs. preterm control; p2 compares term study vs. term control; p3 compares preterm study vs. term study. Pearson's chi-square test or Fisher's exact test was used, as appropriate. NA indicates that a p value was not calculated because both comparison groups had no events.

Table S2. Characteristics of responders and non-responders in the supplementation-response cohort.

Variable	Responders n=55	Non-responders n=4	p
Gestational age, weeks	36.0 [34.0–39.0]	38.5 [37.5–39.0]	0.322
Birth weight, g	2930 [2255–3457]	2879 [2523–3241]	0.845
Baseline 25(OH)D, ng/mL	8.9 [6.8–12.0]	6.8 [5.2–9.8]	0.451
T2 25(OH)D, ng/mL	28.9 [23.3–35.4]	18.9 [18.5–19.3]	<0.001
Δ25(OH)D, ng/mL	18.7 [14.4–26.1]	12.4 [8.7–14.4]	0.019
Baseline DBP, μg/mL	4.7 [3.5–8.2]	5.8 [4.8–6.9]	0.619
T2 DBP, μg/mL	8.4 [6.4–15.9]	14.6 [12.8–16.9]	0.187
ΔDBP, μg/mL	3.0 [0.0–9.9]	8.4 [5.8–11.8]	0.209
Preterm birth	28 (50.9)	1 (25.0)	0.612
Male sex	34 (61.8)	3 (75.0)	1.000
Maternal vitamin D supplementation	12 (21.8)	1 (25.0)	1.000
Maternal limited sun exposure clothing	40 (72.7)	3 (75.0)	1.000
Cesarean delivery	37 (67.3)	2 (50.0)	0.598

Values are presented as median [interquartile range] or n (%). Responders were defined as neonates achieving T2 25(OH)D ≥20 ng/mL after supplementation. Mann–Whitney U test was used for continuous variables, and Fisher's exact test was used for categorical variables. Because only four neonates were classified as non-responders, these analyses were considered exploratory and no multivariable logistic regression was performed.
